# Imbalance of human CD4^+^ T lymphocyte subsets following atrazine treatment

**DOI:** 10.1007/s00204-025-03974-9

**Published:** 2025-03-02

**Authors:** Mahdieh Naghavi Alhosseini, Ambra Maddalon, Luigi Cari, Simona Ronchetti, Graziella Migliorati, Emanuela Corsini, Giuseppe Nocentini

**Affiliations:** 1 Department of Medicine and Surgery, Section of Pharmacology, Università degli Studi di Perugia, Building D, Severi Square 1, 06129 Perugia, Italy; 2 Laboratory of Toxicology, Department of Pharmacological and Biomolecular Sciences, “Rodolfo Paoletti”, Università degli Studi di Milano, Via Balzaretti 9, 20133 Milan, Italy

**Keywords:** Endocrine disruptor, Atrazine, T cell differentiation, Cytokines, Human study, In vitro study

## Abstract

While being banned in the European Union, the herbicide atrazine (ATR) is still one of the most used herbicides in the world. ATR is classified as an endocrine disruptor, but the immunotoxic effects of ATR may also be due to its direct impact on immune cells. To study the effects of ATR on human T cells, we activated T cells present in PBMCs of 8 healthy donors in the presence of ATR (0.1–100 μM). After 4 days of culture, T cells were stained to evaluate cell growth and phenotype by flow cytometry. The results demonstrated that ATR treatment exerts an antiproliferative activity on CD4^+^ T cells and decreases their activation, including the percentage of cytokine-producing CD4^+^ T cells. Among these, the percentage of interferon (IFN)-***γ***- and interleukin (IL)-22-producing CD4^+^ T cells decreased within total CD4^+^ T cells. Moreover, IL-4-, IL-10- and IL-17-producing CD4^+^ T cells decreased within cytokine-producing CD4^+^ T cells. Consequently, ATR caused a dose-related decrease in Th1/Th2 ratio. Many of the effects of ATR treatment were quantitatively different in males and females, with more pronounced effects observed in females. tSNE analysis demonstrated that ATR strongly inhibited the differentiation of two subsets of IFN-***γ***^+^IL-4^+^CD4^+^ T cells from each of the healthy donors tested and promoted greater differentiation of the CD25^+^FoxP3^+^CD4^+^ T cell subset from seven out of the eight healthy donors tested. In conclusion, the study suggests that ATR skews CD4^+^ T cell activation towards Th2, a phenotype that may promote reduced immunosurveillance and increased risk of cancer, as well as Th2-related diseases such as asthma, thereby presenting an environmental and occupation-related risk to human health.

**Graphical abstract:**

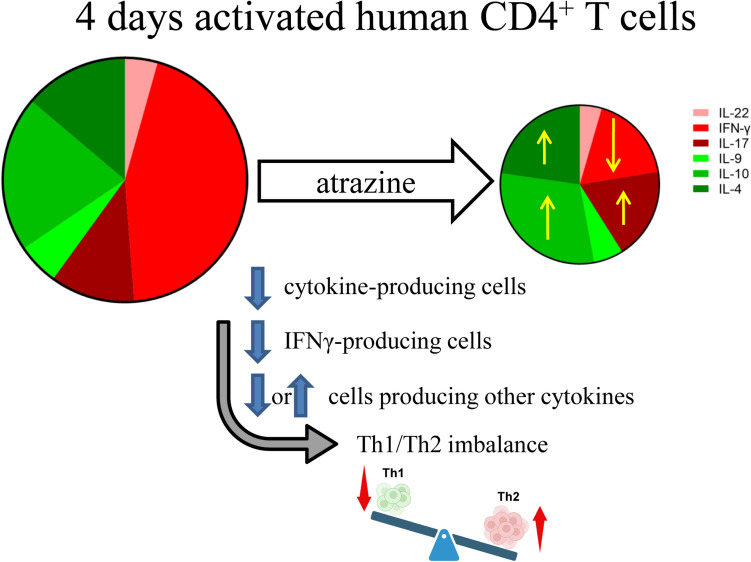

**Supplementary Information:**

The online version contains supplementary material available at 10.1007/s00204-025-03974-9.

## Introduction

The use of herbicides has significantly improved agricultural productivity but it also causes potential hazards to the environment, animals, and human health (Paola Balderrama-Carmona et al. 2020; Tudi et al. 2021). Atrazine (ATR), a broad-spectrum herbicide introduced in the 1950s, remains in use in several countries, including the USA, Australia, China, Iran, Nigeria, and South Africa (Peighambarzadeh et al. 2011; Dabrowski 2015; Olatoye et al. 2021; Galbiati et al. 2021). Due to its long persistence in groundwater (Jablonowski et al. 2011; Syafrudin et al. 2021), it was found one of the most relevant water-contaminating chemicals even across Europe (Loos et al. 2010), despite being forbidden within the European Union for several years before the cited study was performed. ATR contamination has also been detected in animals used for human consumption, with concentrations of 3.4 µM in cow serum and 6–8 nM in catfish fillets (Peighambarzadeh et al. 2011; Olatoye et al. 2021).

ATR is classified as an Endocrine-Disrupting (ED) compound due to its ability to increase estrogen levels by inducing CYP19 (aromatase) transcription (Laville et al. 2006). Exposure to EDs in the early stage of development can alter disease susceptibility later in life and is associated with an increased risk of cancers, autoimmune disorders, and neurological diseases (Schug et al. 2011; Zhao et al. 2013; Galbiati et al. 2021).

It is reasonable that the immunotoxic effects of ATR are due not only to its disruption of endocrine homeostasis but also to its direct impact on immune cells. Indeed, several studies suggest immunosuppressive effects of ATR, including reduced survival and impaired function of CD4^+^ T lymphocytes (Galbiati et al. 2021). For instance, in young male mice, ATR exposure decreases thymus and spleen weights and altered cellular composition (increased CD8^+^ T cells and decreased MHC class II and CD19^+^ cells), and impaired cellular, humoral, and non-specific immune responses (Zhao et al. 2013; Chen et al. 2013). Furthermore, ATR has been shown to inhibit the expression of activation markers CD25 and CD69 in CD4^+^ T cells and modulate serum cytokine levels (Thueson et al. 2015; Ge et al. 2021). Rodent studies suggest that ATR's immunotoxic effects vary by species, age, and sex (Tavalieri et al. 2020; Galbiati et al. 2021), raising concerns about the direct translation of these findings to humans.

Studies on ATR effects on humans are much less. Some epidemiological studies have linked pesticide use in specific regions to increased cancer risk (Thakur et al. 2008; Kaur et al. 2018). However, such studies are complicated by population genetics and co-exposure to other herbicides, insecticides, and lifestyle factors such as alcohol and smoking. In vitro studies on the human Jurkat T-cell line have confirmed findings from rodent models, demonstrating that ATR inhibits T-cell growth and induces apoptosis (Lee et al. 2016). Moreover, in human peripheral blood mononuclear cells (PBMC) treated with low concentrations of mitogens or lipopolysaccharide in a serum-free medium, ATR was shown to reduce the secretion of IFN-γ, IL-5, and TNF-α in the supernatant (Hooghe et al. 2000).

Given the critical role of immune homeostasis in cancer development and autoimmune diseases, our study focuses on the effects of ATR on the differentiation in T-helper (Th) subsets of peripheral human CD4^+^ T cells in primary culture through the study of phenotypical markers and cytokine expression. CD4^+^ T cells were activated in a mixed PBMC culture to evaluate not only the direct effects of ATR on CD4^+^ T cells but also the effects mediated by ATR-exposed PBMCs other than T cells. Our results demonstrate that in proliferating CD4^+^ T cells, ATR induces a concentration-dependent alteration in the percentage of cytokine-expressing cells, leading to an imbalance between Th1 and Th2 subsets.

## Materials and methods

### Atrazine compound

Atrazine (ATR; CAS No. 1912–24-9) was purchased from Sigma-Aldrich (Merck, Darmstadt, Germany). It was dissolved in DMSO, aliquoted, and stored at −70 °C. Dilutions were prepared in culture media as needed.

### Donor demographics

Buffy coat samples from healthy donors were purchased from the Niguarda Hospital in Milan, Italy. All donors were confirmed healthy through clinical and laboratory. Donor demographic characteristics (mean age: 37 years; 4 males, 4 females) are summarized in Table S1. No personal identifiers were collected.

### PBMC isolation and culture

PBMCs were isolated under sterile conditions using Lympholyte® Cell Separation Media (Cedarlane Labs, Canada). Whole blood (5–7 ml) was diluted with PBS, layered onto Lympholyte, and centrifuged at 600 g for 45 min at 4 °C. PBMCs were collected, washed with PBS containing 3% FBS, and resuspended for further use in LDA medium (RPMI supplemented with 10% FBS, 1% penicillin/streptomycin, 1% HEPES, 1% NaPyr, and 1% non-essential amino acids), purchased from Thermo Fisher Scientific, USA. Cells were seeded in 12-well plates at 1 × 10⁶ cells/ml in 2 ml of LDA medium. Dynabeads™ Human T-Activator CD3/CD28 (beads) (Thermo Fisher Scientific) were added at 25 µl per 10⁶ cells. ATR was added at concentrations of 0.1, 1, 10, and 100 µM. Two wells were prepared for each ATR concentration, along with medium-only (CTRL) and DMSO (SCTRL) controls. Cultures were incubated at 37 °C with 5% CO₂ for 4 days.

### Cell counting, staining and flow cytometry

After 4 days, 4 µl of Protein Transport Inhibitor Cocktail (Thermo Fisher Scientific), was added to inhibit cytokine secretion, and cells were incubated for an additional 5 h. Cells were collected, Dynabeads™ magnetically removed, and washed twice with PBS containing 1% FBS.

Cell counts were performed using CountBright™ Absolute Counting Beads (Thermo Fisher Scientific) according to the manufacturer's instructions. In addition to total cell number, cell viability and activation status were assessed.

Surface markers (CD4, CD8, GITR, and CD25) were stained using antibodies diluted 1:200 in PBS with 1% FCS. After staining for 30 min at room temperature, cells were fixed, permeabilized (Foxp3/Transcription Factor Staining Buffer Set, Thermo Fisher Scientific), and stained for intracellular markers (IFN-γ, IL-4, IL-9, IL-10, IL-17, IL-22, Foxp3) for 3.5 h at 4 °C in the dark. The list of used antibodies is reported in Table S2. Cells were then filtered through 70 µm pre-separation filters (Miltenyi Biotec, Germany) and acquired on an Attune NxT flow cytometer (Thermo Fisher Scientific). Data were analyzed using FlowJo V.10.8.1 (BD Biosciences, USA).

### t-SNE analysis of T cell subpopulations

Following conventional analysis, T-cell subpopulations were further investigated using t-distributed stochastic neighbor embedding (t-SNE). Samples were down-sampled to 10,000 cells, concatenated into a single file, and analyzed using the Barnes-Hut t-SNE method with a perplexity of 100 and 1,000 iterations. The results were visualized as 2D t-SNE plots and clusters were defined based on marker expression. Percentages of cells in each cluster were compared across subjects and treatment conditions. FlowJo’s layout editor tool was used to visualize data and generate histograms for phenotypical characterization.

### Statistical analysis

Statistical analyses were performed using Prism 9.5.1 (GraphPad Software, USA). Normality was assessed with Kolmogorov–Smirnov test. Group comparisons were performed using one-way ANOVA or Kruskal–Wallis tests, depending on normality. For repeated measures, RM one-way ANOVA or Friedman tests were applied. Post hoc tests were conducted where appropriate. The two-way ANOVA test was used to evaluate the significant mean difference between LDA-medium/beads-treated control (CTRL) and LDA-medium/DMSO/beads-treated solvent control (SCTRL) in each subject. Results were considered significant at *p* < 0.05, and data are reported as mean ± standard deviation (SD).

## Results

### ATR treatment is not cytotoxic at the tested concentrations

Our study aimed to evaluate the impact of ATR on T-cell differentiation. To exclude any possibility of selection bias, we first assessed its potential cytotoxicity. PBMCs were cultured with anti-CD3/anti-CD28-coated beads to stimulate T-cell activation and expansion. After 4 days, the total number of cells, as well as the number of viable and dead cells, were measured.

**Fig. 1:**
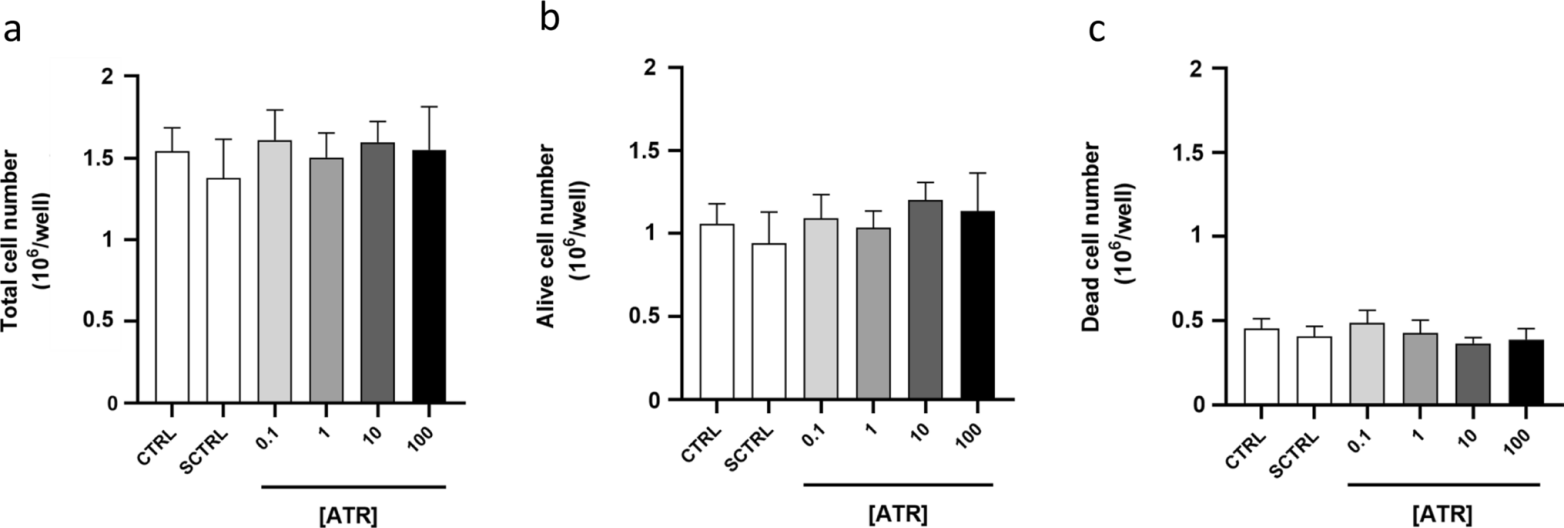
No changes in the number of total cells, alive cells, and dead cells following ATR treatment. The column graphs show the mean** ± **SD of the absolute number of total cells (**a**), alive cells (**b**), and dead cells (**c**) in control (CTRL), solvent control (SCTRL), and ATR-treated samples from eight subjects. Statistical significance between controls and ATR-treated cells was assessed using ordinary one-way ANOVA for normally distributed data and the Kruskal–Wallis test for non-normally distributed data

As shown in Fig. 1, ATR did not significantly affect the total cell count (Fig. 1a), viable cell count (Fig. 1b), or dead cell count (Fig. 1c) compared to the control groups. The relatively high percentage of dead cells (25–30%) across all groups is expected, considering the culture conditions, which primarily provide survival signals to T cells only. Therefore, the observed dead cells likely are B cells and other non-T cells in the PBMC population and is independent of ATR treatment.

### ATR treatment decreases the percentage of activated and cytokine-producing CD4^+^ T cells

Next, we investigated whether ATR modulates the activation and proliferation of T cells, with a focus on CD4^+^ T cells. Flow cytometry analysis of cell size and scattering properties, performed after four days of activation, demonstrated that ATR treatment reduced the activation of T cells. It is well established that activated T cells are larger and have distinct scattering profiles compared to resting T cells (Figure S1). As shown in Figure S2, the percentage of activated cells varied across PBMCs of the healthy donors, likely due to experimental variability or inherent differences in T-cell responsiveness among donors. On the contrary, the percentage of activated cells in CTRL and SCTRL of each donor was very similar, suggesting that the concentration of DMSO used to dissolve ATR did not have any toxic effect.

**Fig. 2:**
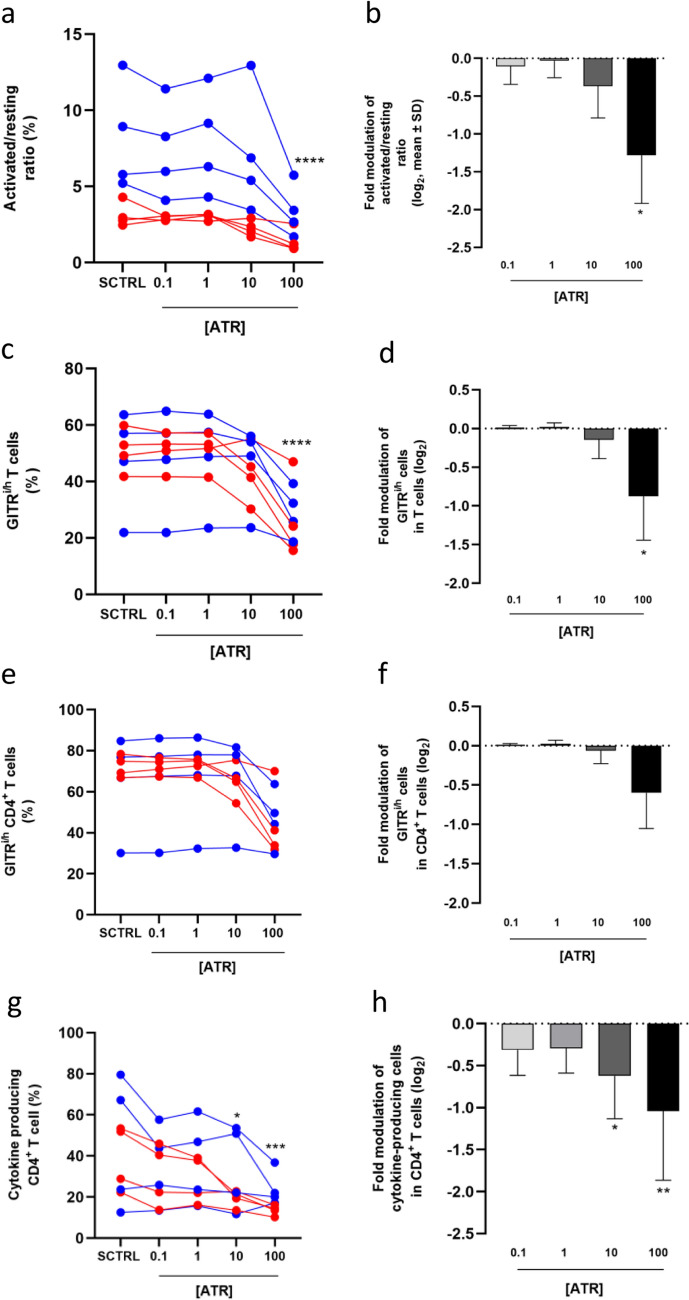
Inhibition of T cell and CD4^+^ T cell proliferation following ATR treatment. The activated/resting cell ratio was assessed in all ATR-treated samples and their respective solvent controls (SCTRL) for each of the 8 subjects (**a**). The mean ± SD of the modulation of the activated/resting cell ratio relative to SCTRL is shown in panel **b**. The percentage of GITR^i/h^ cells was evaluated in the T cell population (**c**) and the CD4^+^ T cell population (**e**), with their respective mean ± SD modulation relative to SCTRL reported in panels **d** and **f**. The percentage of cytokine-producing CD4^+^ T cells is shown in panel **g**, while panel **h** represents the modulation of this percentage compared to SCTRL. Data from male subjects are shown in blue, and data from female subjects in red. Statistical analysis was performed using RM one-way ANOVA (Friedman) for the dot plots (**a**, **c**, **e**, **g**) and Kruskal–Wallis test for the column graphs (**b**, **d**, **f**, **h**)

To quantify ATR's inhibitory effect, the activated/resting cell ratio was evaluated in solvent-treated and ATR-treated cells for each subject (Fig. 2a) and reported as a mean modulation (Fig. 2b). The results indicated a reduction in T-cell activation at 100 µM ATR, with a similar trend observed at 10 µM ATR.

GITR is a marker of T-cell activation. Therefore, we evaluated whether in ATR-treated samples the percentage of T cells and CD4^+^ T cells expressing intermediate to high levels of GITR (GITR^i/h^) were different from that of SCTRL cells (Figs. 2c and 2e). In Figs. 2d and 2f, the percentage of GITR^i/h^ cells in treated samples was divided by the percentage of GITR^i/h^ cells in solvent-treated samples to evaluate the modulation, and the mean modulation is reported as log2. The results showed that following ATR treatment the percentage of GITR^i/h^ cells decreased by 30–50%, significantly (T cells) and non-significantly (CD4^+^ T cells) at 100 µM ATR (Fig. 2c–f). These data further support the conclusion that ATR inhibits T-cell and CD4^+^ T-cell activation in a dose-dependent manner.

Upon T-cell receptor (TCR) and CD28 stimulation, resting CD4^+^ T cells differentiate into cytokine-producing helper T-cell subsets. Therefore, after 4 days of PBMC culture with anti-CD3/anti-CD28-coated beads, we evaluated the percentage of cells producing one or more T helper key cytokines (IFN-γ, IL-4, IL-10, IL-17, IL-22, and IL-9) in ATR-treated CD4^+^ T cells. Figures 2g and 2h and Table S3 show that the percentage of cytokine-producing cells was lower in ATR-treated samples as compared to SCTRL. The effect was dose-dependent, with a decrease of 10–39% observed even at the lowest ATR concentration (0.1 µM) in six out of eight donors (Table S4). Notably, the effect on cytokine-producing CD4^+^ T cells was more pronounced in PBMC from female donors at all the ATR concentrations tested and the decrease was significant in most of the concentrations tested (Table S4). In contrast, the decrease in male samples was not statistically significant, likely due to variability in control activation levels (in particular donors #1 and #8)(Table S3).

### ATR decreases the percentage of CD4^+^ T cells

Given ATR's inhibitory effects on CD4^+^ T cell activation, even CD4^+^ T cell proliferation and T cell number might have been affected. Indeed, ATR exposure significantly reduced the percentage of CD4^+^ T cells, with an 8% decrease observed at 100 µM ATR (*p* = 0.0007) compared to SCTRL (Tables S5 and S6). When analyzed by sex, both male and female groups exhibited significant reductions in CD4^+^ T cell percentages at 100 µM ATR (*p* = 0.027 for males, *p* = 0.040 for females). These findings suggest that ATR-mediated inhibition of CD4^+^ T cell activation leads to a decrease in CD4^+^ T cell proliferation.

### ATR reduces the percentage of INFγ- and IL-22-producing cells

To understand how ATR decreases CD4^+^ T cell activation, we evaluated the effect of ATR on the percentage of CD4^+^ cells expressing IFN-γ, IL-4, and IL-10 cytokines produced, even not exclusively, by Th1 (IFN-γ) and Th2 (IL-4 and IL-10) cell subsets, and other cytokines (IL-17, IL-22, and IL-9) produced, even not exclusively, by other T helper subsets such as Th17, Th22, and Th9.

**Fig. 3:**
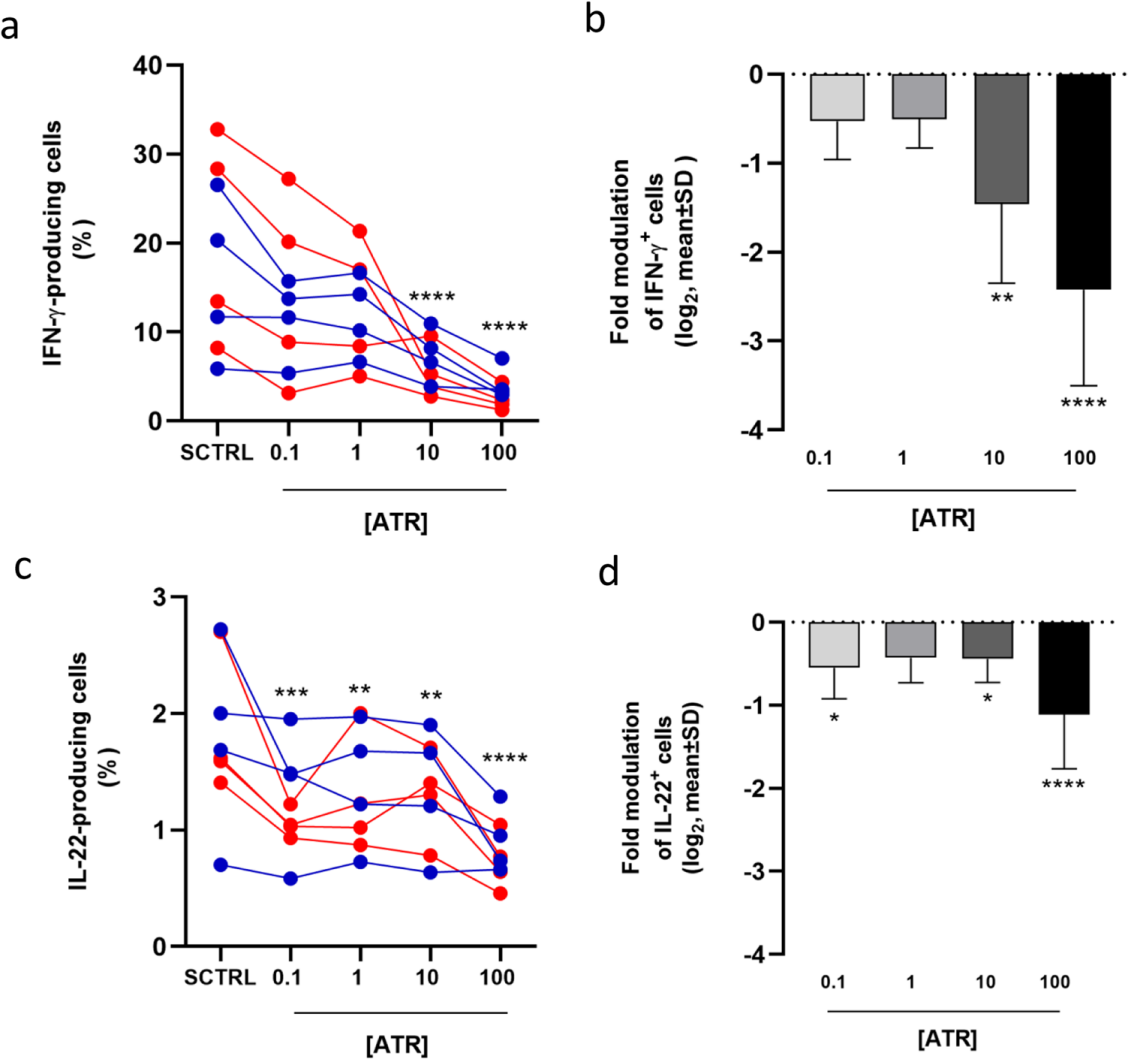
Decreased percentage of IFN-γ and IL-22-producing CD4^+^ T cells following ATR treatment. The percentage of IFN-γ (**a**) and IL-22 (**c**) producing cells was assessed in all ATR-treated samples and their respective solvent controls (SCTRL) for each of the 8 subjects. The mean ± SD of the modulation of IFN-γ (**b**) and IL-22 (**d**) producing cells, relative to SCTRL is shown. Data from male subjects are shown in blue, and data from female subjects in red. Statistical analysis was performed using RM one-way ANOVA (Friedman) for the dot plots (**a**, **c**) and Kruskal–Wallis test for the column graphs (**b**, **d**)

Data show that ATR treatment significantly decreases the percentage of IFN-γ- and IL-22-producing CD4^+^ T cells in a dose-dependent manner compared to SCTRL (Figs. 3 and S3). The reduction of IFN-γ-producing cells is relevant at 10 and 100 µM ATR, with a 3- to fivefold decrease, respectively, and a trend toward reduction is observed even at lower concentrations. PBMCs from females appear to be more responsive to inhibition than PBMCs from males (Figure S3). The decrease of IL-22-producing cells is lower in magnitude (1.5- to twofold) but is significant even at 0.1 µM ATR. No significant modulation was observed for cells producing other cytokines, despite a general downward trend (Figure S4). In summary, two out of the three investigated pro-inflammatory cytokines are expressed by a lower percentage of cells following ATR treatment.

### ATR modulates the cytokine-producing cells favoring a Th2-related phenotype

**Fig. 4:**
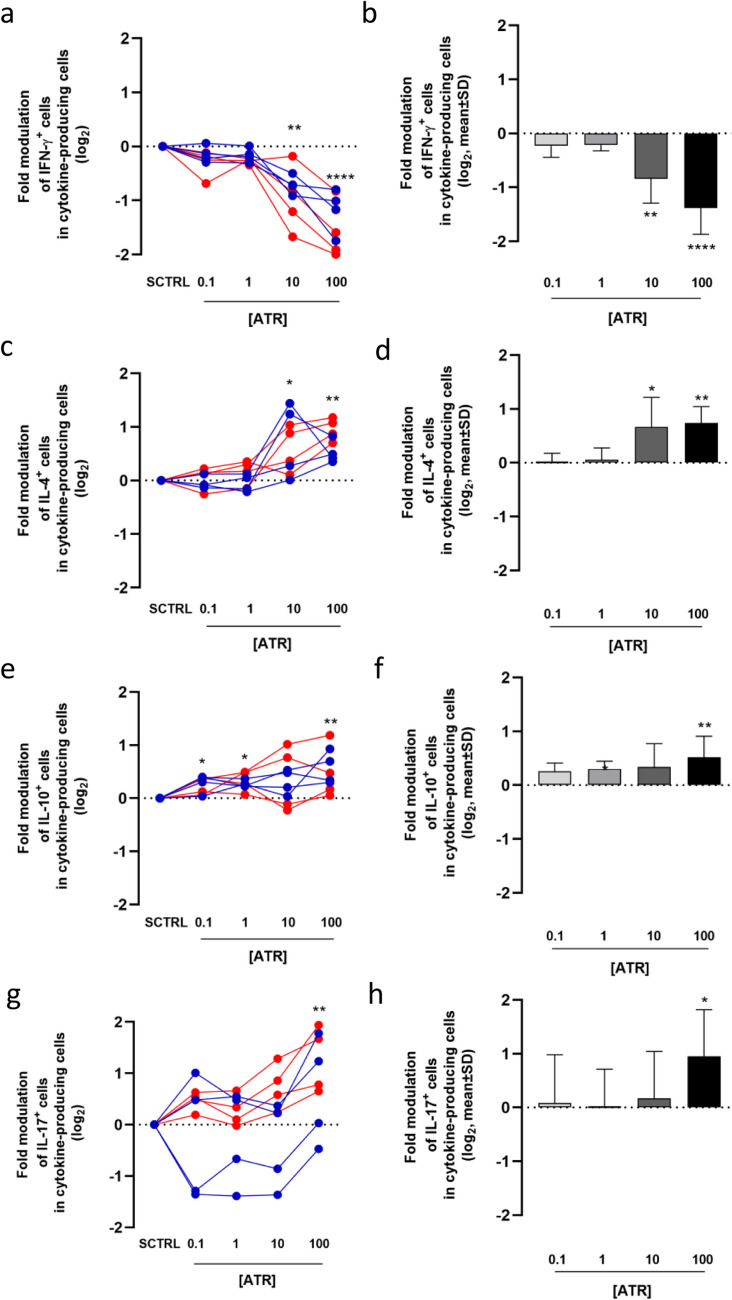
Modulation of IFN-γ, IL-4, IL-10 and IL-17-producing cells among cytokine-producing CD4^+^ T cells following ATR treatment. Among cytokine-producing cells, the modulation of IFN-γ (**a**), IL-4 (**c**), IL-10 (**e**), and IL17 (**g**) producing cells, relative to SCTRL is shown for each of the eight subjects. The mean ± SD of the modulation of IFN-γ (**b**), IL-4 (**d**), IL-10 (**f**), and IL17 (**h**) producing cells is also reported. Data from male subjects are shown in blue, and data from female subjects in red. Statistical analysis was performed using RM one-way ANOVA (Friedman) for the dot plots (**a**, **c**, **e**, **g**) and Kruskal–Wallis test for the column graphs (**b**, **d**, **f**, **h**)

Since, in vivo, only cytokine-producing cells contribute to a pro-inflammatory, pro-allergic, or suppressive microenvironment, we re-analyzed the data evaluating exclusively cytokine-producing cells and cytokines were roughly considered as pro-inflammatory (IFN-γ, IL-17, IL-22) and anti-inflammatory/pro-allergic (IL-4, IL-10, IL-9). In Figure S5 we represented the cells that produce pro-inflammatory cytokines with different shades of red and the cells that produce inhibitory/pro-allergic cytokines with different shades of green. Interestingly, in SCTRLs, male-derived CD4^+^ T cells exhibited a balance between pro- and anti-inflammatory cytokine producers. In contrast, female-derived CD4^+^ T cells showed a bias towards pro-inflammatory cytokine production (Th1 cells). We investigated whether ATR treatment changes the cytokine balance within the cytokine-producing cells of each donor, using the solvent-treated sample of PBMC from the same donor as a control. Not surprisingly, the percentage of IFN-γ producing T cells decreased within the cytokine-producing T cell population (Fig. 4a–b). The decrease was observed only at 10 and 100 μM ATR concentrations in both males and females (Figure S6).

Interestingly, the results showed an upward trend for IL-4-, IL-10-, and IL-17-producing cells in almost every sample (Fig. 4c, e, and g), particularly at higher ATR concentrations (Fig. 4d, f, and h). The increase in IL-17-producing cells was more pronounced in female-derived samples, reaching significance at 100 µM ATR (Figs. 4g and S7). In this analysis, the percentage of IL-9- and IL-22-producing cells was not modulated by ATR treatment (Figure S8).

**Fig. 5:**
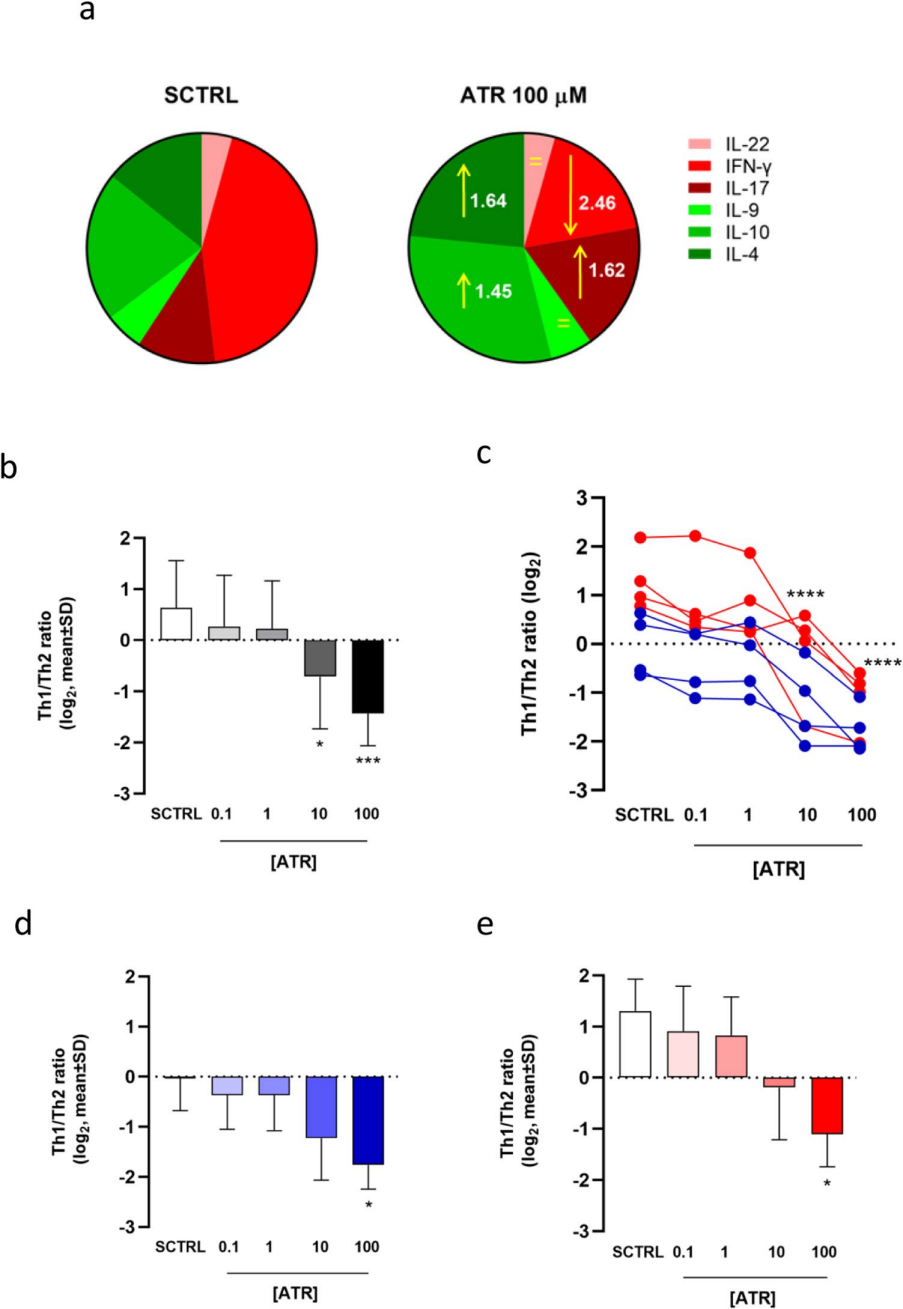
Decrease of Th1/Th2 ratio following ATR treatment. Panel (**a**) shows pie charts of solvent control (SCTRL, left) and ATR-treated (100 μM, right) samples, illustrating the overall modulatory effects of ATR on cells expressing individual cytokines among cytokine-producing cells. Anti-inflammatory cytokines (IL-9, IL-10, and IL-4) are represented in green, while pro-inflammatory cytokines (IL-22, IFN-γ, and IL-17) are shown in red. Upward arrows indicate an increase and downward arrows indicate a decrease in the relative amount of each cytokine. The length of the arrows reflects the level of modulation, and the mean fold change in ATR-treated samples compared to SCTRL is reported within the pie charts. Panel (**b**) reports the Th1/Th2 immune response ratio as mean ± SD, while panel (**c**) displays this ratio for each of the eight subjects individually. Data from male subjects are shown in blue, and data from female subjects in red. Panels (**d**) and (**e**) present the mean ± SD Th1/Th2 immune response ratio for male and female subjects, respectively. Statistical analysis was performed using RM one-way ANOVA (Friedman) for the dot plot (**c**) and Kruskal–Wallis test for the column graphs (**b**, **d**, **e**) (colour figure online)

Figure 5a and Figures S9 and S10 summarize the overall effects of ATR on cells expressing a single cytokine among the cytokine-producing cells. At the highest concentration tested, ATR increases two out of three kinds of cells expressing pro-allergic/regulatory cytokines (IL-4 and IL-10) while simultaneously reducing the percentage of cells producing IFN-γ, the key pro-inflammatory cytokine secreted by Th1 cells. Moreover, ATR slightly increases the percentage of cells expressing IL-17, a pro-inflammatory cytokine mainly expressed by Th17, particularly in females.

To evaluate the Th1/Th2 ratio of immune responses following ATR treatment, we considered the IFN-γ^+^ cells to represent Th1 responses and IL-4^+^/IL-10^+^ cells to represent Th2 responses. As shown in Fig. 5b, ATR treatment dose-dependently decreases the Th1/Th2 ratio, suggesting that ATR shifts CD4^+^ T cell activation towards a Th2-dominant response. The decrease of Th1/Th2 ratio is relevant and significant following 10 and 100 μM ATR treatments, with similar significance observed when males and females were analyzed separately (Fig. 5c–e).

### The shift towards Th2-related phenotype is confirmed by tSNE analysis

**Fig. 6:**
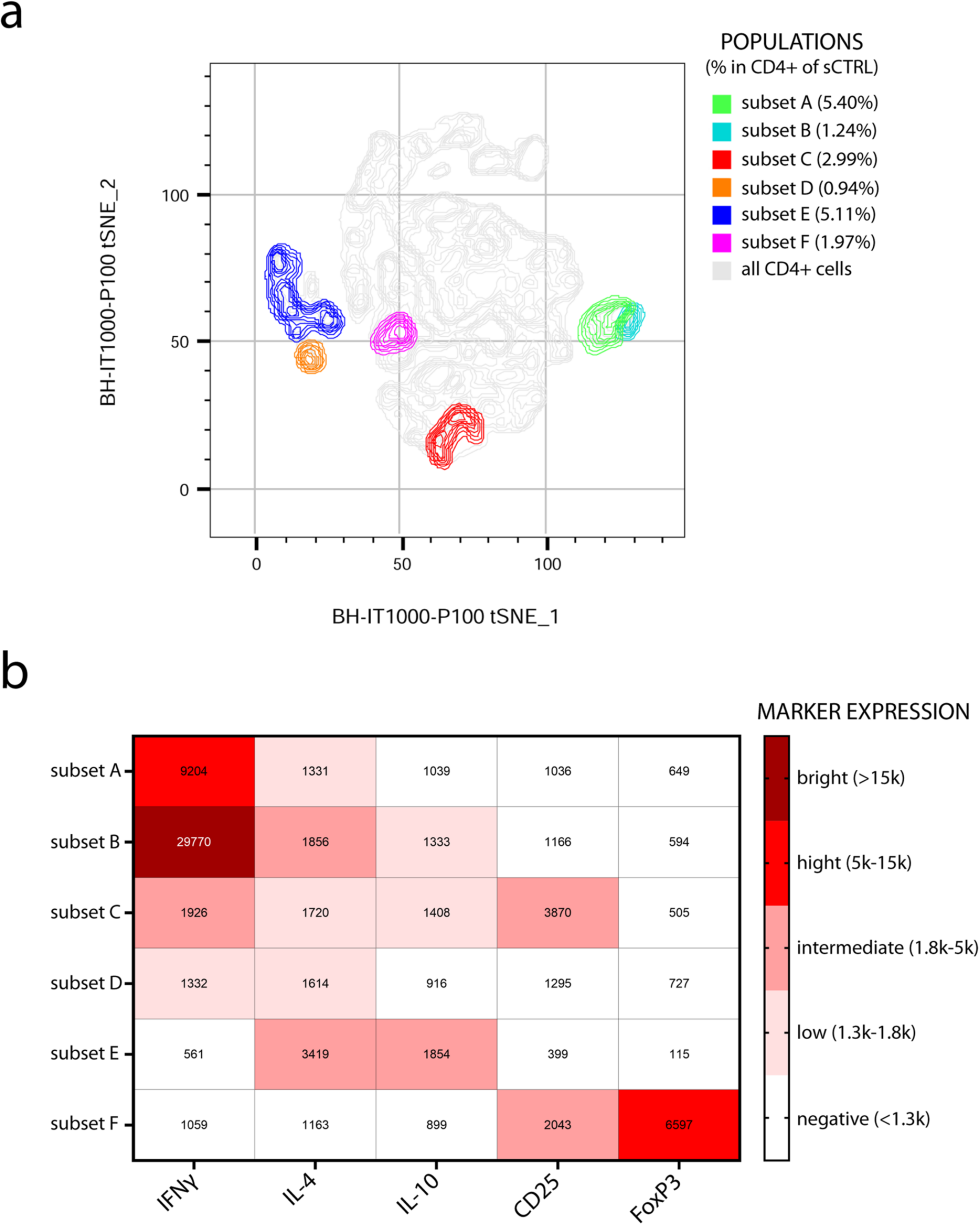
tSNE analysis of CD4^+^ T cells following T cell activation. Panel (**a**) displays the 2D t-SNE plot of CD4^+^ T cell, with subsets of cells expressing key markers (IFN-γ, IL-4, IL-10, CD25, and FoxP3) highlighted in different colors. The total population of CD4^+^ T cells is shown in grey for comparison. Cell subsets representing less than 0.8% of the total CD4^+^ T cells were excluded from the analysis. Panel (**b**) provides the phenotypical characterization of each CD4^+^ T cell subset, with a heatmap reporting the mean fluorescence intensity (MFI) of each marker across the different subsets (colour figure online)

To explore the ATR-induced imbalance in CD4^+^ T cell differentiation from another perspective, we performed a tSNE analysis focusing on the subsets expressing IFN-γ, IL-4, IL-10, and FoxP3 (Fig. 6a). After merging subsets with similar phenotypes (adjacent on the tSNE plot) and excluding those representing less than 0.8% of CD4^+^ T cells that could not be merged, six distinct subsets were identified: three expressing mainly IFN-γ (subsets A-C), one expressing low levels of IFN-γ and IL-4 (subset D), one expressing IL-4 and IL-10 (subset E), and one expressing CD25 and FoxP3 (subset F)(Fig. 6b).

**Fig. 7:**
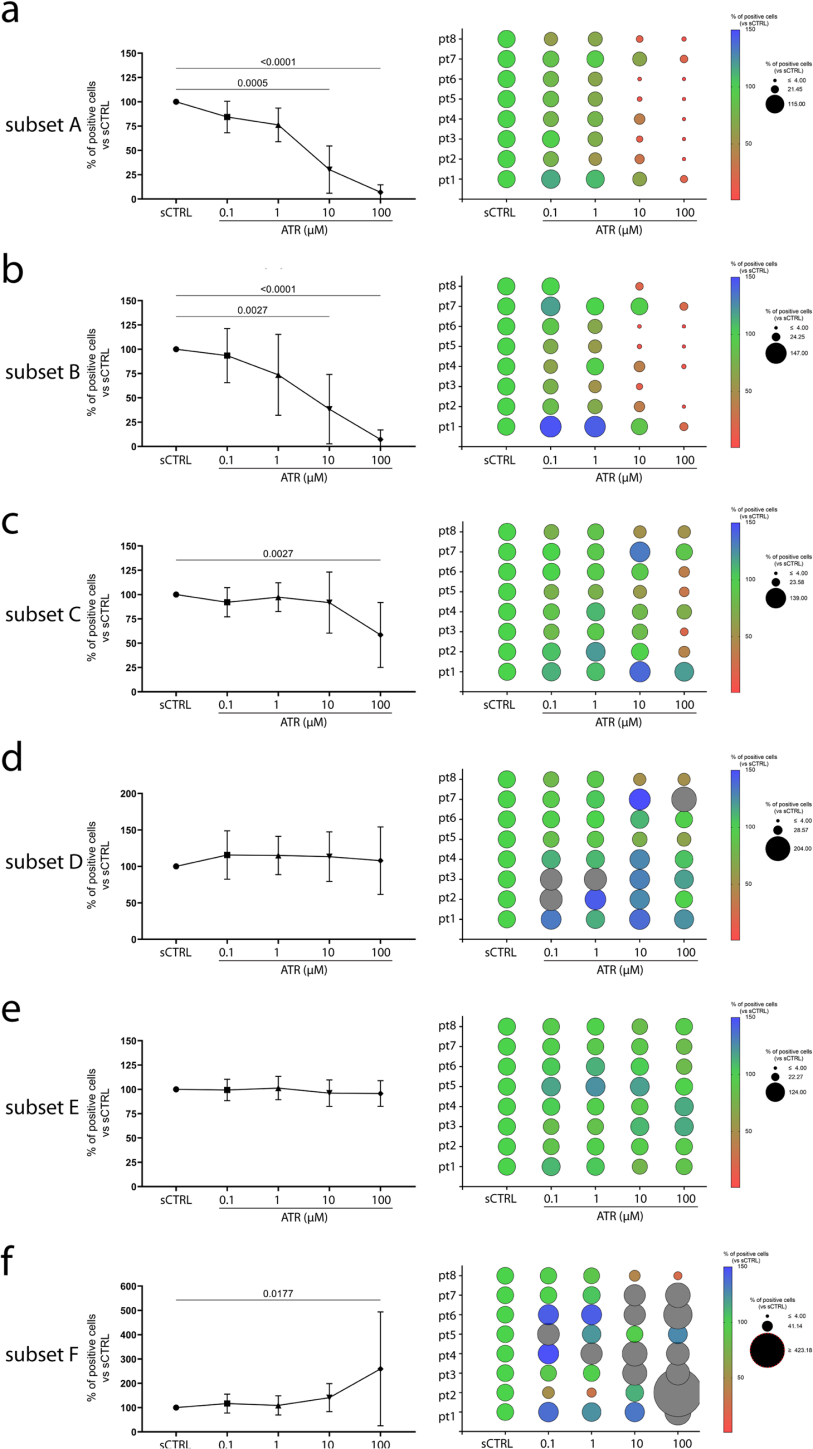
ATR-dependent changes of the percentage of CD4^+^ T cell subsets defined by tSNE analysis. On the left, panels (**a**), (**b**), (**c**), (**d**), (**e**), and (**f**) show the mean percentage of CD4^+^ T cell subsets A, B, C, D, E, and F, respectively, in ATR-treated samples compared to solvent control (SCTRL, set to 100%). On the right, panels show bubble plots representing individual data of the eight subjects; the size of each bubble indicates the magnitude of modulation, and the red–green–blue color scale reflects the direction and intensity of change. Statistical analysis was performed using RM one-way ANOVA (Friedman)

The two subsets expressing high and very high levels of IFN-γ (subsets A and B) almost disappear following treatment with 100 µM ATR, with a significant reduction even at 10 µM ATR in most subjects tested (Fig. 7a–b). Notably, subset A is the most frequent IFN-γ^+^ subset (more than 5% of CD4^+^ T cells). Subset B, representing 1–1.5% of CD4^+^ T cells, expresses very high levels of IFN-γ and can be labeled as IL-4-producing, FoxP3^−^ Th1 cells. Subset C (IFN-γ^+^CD25^+^FoxP3^−^) consisting of fully activated CD4^+^ T cells, represents about 3% of CD4^+^ T cells and decreases following 100 µM ATR treatment (Fig. 7c). The decrease of subsets A, B and C reasonably accounts for most of the decrease of IFN-γ^+^ cells shown in Fig. 3a, as these subsets represent about 10% of CD4^+^ T cells. In contrast, the cells in subset D, which produce low levels of IFN-γ are about 1% of CD4^+^ T cells and did not decrease following ATR treatment (Fig. 7d).

Subset E, comprising about 5% of CD4^+^ T cells, exclusively expresses IL-4 and IL-10 confirming that in our experimental system, most IL-4-expressing cells also co-express IL-10, representing cells differentiated toward Th2 phenotype (Fig. 7e). Their percentage remains unchanged following ATR treatment, confirming that Th2 differentiation is not affected by ATR. However, when considered alongside the marked reduction in IFN-γ^+^ cells, it is evident that ATR skews the balance towards Th2 cells.

Th2 cells play roles in both allergic disease development and immune regulation through IL-10 production. To understand whether the ATR-induced Th2 phenotype favors a pro-allergic or immunosuppressive response, we investigated the effect of ATR on the differentiation/expansion of FoxP3^+^ Treg, represented by subset F, which contains CD25^+^FoxP3^+^ cells. Subset F constitutes approximately 2% of CD4^+^ T cells and doubled following 100 µM ATR treatment (Fig. 7f). Interestingly, the effects were quite different between donors. In one subject (#8), the percentage of subset F cells decreased in a dose-dependent manner, while in two subjects (#3 and #4), the percentage peaked following 10 µM ATR treatment. In subject #5, the percentage of cells peaked following 0.1 µM ATR treatment. In the remaining four subjects, the peak was observed following 100 µM ATR treatment. These results suggest that, although there is a general trend of increased FoxP3^+^ Treg cells at higher doses of ATR, the effect varies between individuals, potentially due to differences in immune status or co-exposure to other factors.

## Discussion

Our in vitro study on activated T cells from human PBMCs demonstrates that ATR has several effects on CD4^+^ T cells including inhibition of activation and proliferation, a reduction in the number of IFN-γ- and IL-22-producing cells, a Th1/Th2 imbalance towards the Th2 phenotype and subject-specific effects on the differentiation/expansion of Treg cells. ATR modulates CD4^+^ T cell activity in both males and females, with a more pronounced effect in females, confirming our previous results demonstrating sex-dependent effect of endocrine disruptors (Maddalon et al. 2023).

### ATR-dependent decrease of IFN-γ- and IL-22-producing cells

One of the main findings of our study is the ATR-dependent decrease of IFN-γ- and IL-22-producing CD4^+^ T cells. The reduction of IL-22-producing cells is significant even at the lowest ATR concentration tested (0.1 µM). tSNE analysis identified four major subsets of IFN-γ-producing CD4^+^ T cells (subsets A, B, C, and D), with the decrease of subsets A, B, and C following ATR treatment. Interestingly, subsets A and B almost disappear in the PBMC of several subjects following 10 and 100 µM ATR treatment. Considering the high IFN-γ expression in these subsets, it is likely that the levels of IFN-γ in the culture media may be considerably lower in ATR-treated cells compared to controls. Indeed, Hooghe et al. reported a 70% reduction of IFN-γ in culture media following PBMC treatment with 3 µM ATR under conditions similar to ours (PBMCs activated for 2 days by mitogens in a serum-free medium)(Hooghe et al. 2000). ATR-dependent decrease of IFN-γ has been observed in both in vivo and in vitro studies on mice (Chen et al. 2013; Wang et al. 2023). Holásková et al. investigated the long-term effects of perinatal ATR exposure on the neonatal immune system in mice and found a reduction in IFN-γ production by activated T cells in male mice, but not in females, at 6 months of age, suggesting a biased differentiation of CD4^+^ T cells determined by perinatal ATR exposure (Holásková et al. 2019). We also show different effects of ATR on IFN-γ-producing CD4^+^ T cells from males and females but, in our study, the effects on females are more relevant.

To our knowledge, the effect of ATR on IL-22 production has not been previously demonstrated. Although our results suggest a reduction in IL-22-producing cells, tSNE analysis did not identify a specific region grouping these cells, suggesting that IL-22-producing cells do not belong to a clearly defined subpopulation.

### ATR-dependent relative increase of IL-10-and IL-4-producing cells

Our study demonstrates an ATR-dependent increase of IL-10 and IL-4-producing CD4^+^ T cells among the cytokine-producing population. The effect was observed when considering the percentage of IL-10 and IL-4-producing CD4^+^ T cells within the cytokine-producing cells (Fig. 4) and not when considering the percentage of IL-10 and IL-4-producing CD4^+^ T cells within CD4^+^ T cells (Figure S4 and tSNE analysis). However, in our opinion, the former result is more relevant than the latter, because the pool of cytokines in the tissue microenvironment depends on activated T cells.

Previous studies in mice have reported a reduction in IL-10 release/content in the brain following ATR exposure, however, the specific cell types responsible of IL-10 production were not investigated (Genovese et al. 2021; Dai et al. 2022). In contrast, subcutaneous administration of ATR inhibited IL-6 and IL-12 production in the peritoneal cavity, while IL-10 levels either increased or remained unchanged (Pruett et al. 2006). These findings suggest that ATR's effects on IL-10 may vary depending on the tissue studied.

An in vitro study by Devos et al. did not find an increase of IL-10 in ATR-treated PBMC but did not report the percentage of IL-10-expressing cells (Devos et al. 2004). Thus, bona fide, our study is the first to demonstrate a relative increase in IL-10-producing T cells in human PBMCs.

In a study on mice, ATR exposure affects 4T1 breast cancer development, and one of the findings was that IL-4 was increased in the serum and tumor microenvironment (Wang et al. 2023). Similarly, ATR treatment in rats was shown to elevate serum IL-4 levels (Ge et al. 2021). Our findings suggest that ATR has comparable effects on IL-4 production in humans as those observed in rodents.

### ATR-dependent increase of FoxP3^+^ Treg cells

In vitro activation of murine T cells in the presence of ATR resulted in a threefold increase in the frequency of Foxp3^+^CD4^+^ T cells (Thueson et al. 2015). A more recent study on mice bearing 4T1 breast cancer also reported an increase in tumor-infiltrating Tregs following 28 days of ATR treatment (Wang et al. 2023). In our study, we observed a significant increase of FoxP3^+^ Tregs at 100 µM ATR, a concentration similar to that tested in the above-mentioned mice. However, this concentration is unlikely to be reached in human tissues under real-world conditions (see paragraph below). Therefore, it is reasonable to conclude that in most individuals, the levels of ATR encountered in daily life would not result in an increase in Tregs. Nonetheless, in the PBMC from three subjects, we observed an unexpected dose-independent increase of Treg at lower ATR concentrations (10 µM and 0.1 µM). Further studies must verify whether this result is a laboratory artifact or if ATR has dose-independent effects on Tregs in certain individuals, depending on genetics and/or environmental factors.

A study in rats enforces the idea that ATR’s effects on Tregs may vary depending on dosage and other factors and are not modulated linearly. Indeed, in animals treated with ATR over several weeks, authors observed that low doses led to a decrease in Treg cells, while higher doses increased Tregs (Ge et al. 2021).

### Relevance of our data for effects on living beings: studies on animals and human volunteers evaluating ATR levels in the human body

Data on the ATR concentration in tissues/organs of individuals living in ATR-polluted environments are not available, but studies in animals and human volunteers provide some insights. An old study using radiolabeled ATR in rats suggests that ATR and/or its metabolites tend to accumulate in tissues (Bakke et al. 1972). Indeed, in rats administered with one dose of radiolabeled ATR and sacrificed after 2, 4, or 8 days, the ATR concentration in the brain and muscles after 2 days was equal to 1.6 and 0.6 ppm, respectively, and after 8 days was equal to 1.1 and 0.5 ppm, respectively. Therefore, data suggest that in the brain the tissue half-life of ATR is about 11 days and in the muscle is 20–25 days, and that a relevant accumulation may be observed if ATR is administered daily. The same study demonstrated a body retention of about 15% of the ATR (and/or its metabolites) 72 h after the administration by oral route. In a more recent study on human volunteers, about 8% of applied ATR was retained in the body seven days after application to the skin (Gilman et al. 1998). These studies suggest a very high affinity of ATR and/or its metabolites for mammalian tissues, possibly suggesting the potential for accumulation following repeated exposure.

A recent study evaluated the ATR concentration in fish feed and catfish fillets from commercial aquaculture farms in Southwestern Nigeria (Olatoye et al. 2021). In both fish feed and catfish fillets, the ATR concentration was about 1.5 µg/kg, demonstrating that in these fishes ATR accumulates in muscles, confirming the above-mentioned studies in mammals. Similarly, in cows fed with corn silage from Iranian farms in which ATR was used at 3 kg/hectare, ATR levels in serum reached 3.4 µM (Peighambarzadeh et al. 2011). Unfortunately, the authors did not measure the ATR concentration in the muscles of these cows but we can suppose from the above-reported study the ATR concentration in the muscles may be equal to or even higher than that of the serum.

In summary, these studies suggest that ATR can accumulate in the tissues following repeated exposure and that in some cases, tissue concentrations could reach 10 µM or higher in some parts of the body.

### Relevance of our data for effects on living beings: studies evaluating ATR levels in the human body following ATR spread

Several studies have measured ATR concentration in the urine of agricultural workers experiencing occupational exposure to ATR. Koivunen et al., reported ATR levels above 0.3 µM in the urine of the most highly exposed farmers (Koivunen et al. 2006).

Denovan et al. evaluated the concentration of ATR in the saliva of farmers on the day they applied the ATR, and the mean concentration was 0.17 µM (Denovan et al. 2000). According to a study in mice, the ATR concentration in the saliva is 66% of the ATR concentration in the serum (Chensheng Lu Leigh C. Anderson Rich 1997), so the mean serum concentration in these farmers would likely exceed 0.2 µM.

In a comprehensive review on ATR, authors write that, for farmers working on field corn, a mixer-loader-tender-applicator would be exposed to 2.8 mg per person after a day's work (Gammon et al. 2005). Based on dermal absorption of 5.6%, this translated into an absorbed daily dosage of 1.8–6.1 µg/kg^−1^ body weight day^−1^ corresponding to 8–28 nmol kg^−1^ body weight day^−1^. For example, if considering a mean absorbed daily dosage of 20 nmol kg^−1^ body weight day^−1^ and a body weight of 70 kg, a person would absorb 1.4 µmol day^−1^ which could mean tissue retention of about 0.1 µmol every day with a likely 5–10 µmol ATR deposition in tissues after 1–3 months of daily exposure.

Summarizing, these findings suggest that in the worst cases, ATR concentration in serum may reach 0.1–0.2 µM, with tissue concentrations potentially reaching 1–10 µM in selected tissues. One study suggests that the exposition of children living with farmers using ATR may be similar (Adgate et al. 2001).

### Relevance of our data for effects on living beings: the difference between in vitro and in vivo studies

It is well known that in vitro studies offer a simplified model for studying the effects of substances. In the case of immune system studies, factors such as cell recirculation and cytokine signaling in specific microenvironments are not accounted for. While we chose to study ATR's effects on T cells within PBMC cultures instead of purified T cells to include a broader cytokine environment, this approach still does not fully mimic in vivo conditions. We know that, in this way, we avoided just one of the several factors making in vitro testing a biased way to test the effects of substances. Another limitation of our study is that we tested only the ATR effects whereas, in real-life scenarios, multiple pollutants may be present simultaneously. Finally, the number of tested samples is low, so decreasing the likelihood of obtaining significant data and certain ages (e.g. children or newborns) may be more likely to be sensitive to ATR, and we did not test PBMC from this kind of subjects.

In our study, several effects on ATR-treated PBMC were observed at 100 µM ATR concentration and most of them were detectable even at 10 µM ATR concentration. In a few evaluations, effects were observed even at lower concentrations, with a clear dose-dependent effect. Can the effect observed following 10 µM ATR treatment be useful only to predict the in vivo effect for the few farmers, if any, reaching 10 µM ATR concentration in some of their tissues? Some reasons let us hypothesize that the effects observed following 10 µM ATR treatment may concern several people living in polluted areas. First, we performed an in vitro study on PBMC treated for 4 days with ATR. When testing the toxicity of potential anticancer drugs by in vitro studies, it has been supposed and verified that the drug effects are proportional to the time of contact so that if the drug is toxic at 72 µM after 1 h of contact it is supposed that it has a similar effect at 1 µM after 72 h of contact. In the hypothesis that the time-dependent effect is verified even for longer exposure times, if a human is exposed for 3 months at 1 µM ATR, the observed effects may be those observed following 20 μM exposure for 4 days. A second reason is the variability of the observed effects within people. Genetic and/or epigenetic differences may make people more or less sensitive to the ATR effect, decreasing the likelihood of obtaining significant data and making it possible that some people are more sensitive than others. Finally, differentiation of the cells of the immune system is a long-lasting event determined by the balance of several environmental factors. It is possible to hypothesize that exposure to low concentrations of ATR determines effects that can be considered irrelevant but that favor long-term changes such as, for example, Th1/Th2 imbalance several years after the exposure to ATR.

### Potential ATR-dependent Th1/Th2 imbalance in humans

If the tissue concentrations of ATR in subjects living in ATR-polluted areas are equal to 1–10 µM, our study predicts a Th1/Th2 imbalance favoring Th2-related diseases such as asthma and atopy, particularly in children and young adults with developing immune systems. Indeed, in a study evaluating the chemical predictors of wheeze among farmer pesticide applicators, applying ATR more than 20 days/year, ATR exposure was associated with an increased risk of wheezing, with an OR of 1.5 (95% CI 1.2–1.9)(Hoppin et al. 2002). Although our study did not directly demonstrate a link between ATR and increased atopy prevalence, the possibility cannot be excluded.

If the Th1/Th2 imbalance is accompanied by inhibition of T cell activation and an increased percentage of Tregs, our study predicts a decreased immunosurveillance of the immune system and an increased probability of cancers in the long term. Indeed, a just-published epidemiologic study evaluated the ATR-cancer incidence associations among pesticide applicators and demonstrated suggestive associations with some malignancies in overall, age-specific, and lagged analyses (Remigio et al. 2024), confirming previous epidemiological studies (Hoar Zahm et al. 1993; Andreotti et al. 2020). In particular, ATR increased the risk of non-Hodgkin lymphoma, soft tissue sarcoma, kidney cancer, and aggressive prostate cancer in the highest quartile among men aged less than 60 years old. Some experimental and epidemiological studies suggest that ATR increases the risk of breast cancer (Pintér et al. 1990; Kettles et al. 1997; Wang et al. 2023). The ATR-dependent increased risk of endocrine-related cancer and/or their aggressiveness may be due not only to the imbalance of T cell subsets, as supposed in the present study, but also to the well-described activity of endocrine disruption by ATR (Galbiati et al. 2021; Remigio et al. 2024).

## Conclusions

Our study on human T cell differentiation confirms that ATR exerts antiproliferative effects and inhibits T cell activation. These effects are determined by the ATR-dependent reduction of IFN-γ which results from a decreased differentiation/proliferation of cells producing high levels of IFN-γ. The relative increase of IL-4- and IL-10-producing cells as compared to IFN-γ-producing cells leads to a relevant imbalance in the Th1/Th2 ratio, favoring Th2 cells. This shift, whether or not associated with an increase in Treg cell percentages, might favor decreased immunosurveillance and increased cancer risk, as well as Th2-related diseases such as asthma, as demonstrated by epidemiological studies on farmers or ATR-producing workers. On the contrary, our study does not provide any evidence to draw conclusions regarding the potential effects of low ATR levels contaminating soil, tap water, and food.

## Supplementary Information

Below is the link to the electronic supplementary material.

Supplementary file1 (PDF 10320 KB)

## Data Availability

The datasets generated during and/or analysed during the current study are available from the corresponding author on reasonable request.
