# Supplementary material for: Imbalance of human CD4+ T lymphocyte subsets following atrazine treatment
Source: Arch Toxicol. 2025 Mar 2;99(4):1455–69. doi: 10.1007/s00204-025-03974-9 (PMC11968556; doi:10.1007/s00204-025-03974-9)
Supplement: Supplementary file 1 — Supplementary file1 (PDF 10320 KB) [file 204_2025_3974_MOESM1_ESM.pdf]

Imbalance of human CD4$^+$ T lymphocyte subsets following atrazine treatment

Mahdieh Naghavi Alhosseini[1], Ambra Maddalon[2], Luigi Cari[1#], Simona Ronchetti[1], Graziella Migliorati[1], Emanuela Corsini[2], Giuseppe Nocentini[1]

[1]Department of Medicine and Surgery, Section of Pharmacology, Università degli Studi di Perugia, 06129, Perugia, Italy

[2]Laboratory of Toxicology, Department of Pharmacological and Biomolecular Sciences 'Rodolfo Paoletti', Università degli Studi di Milano, Via Balzaretti 9, 20133, Milan, Italy

Supplementary Information

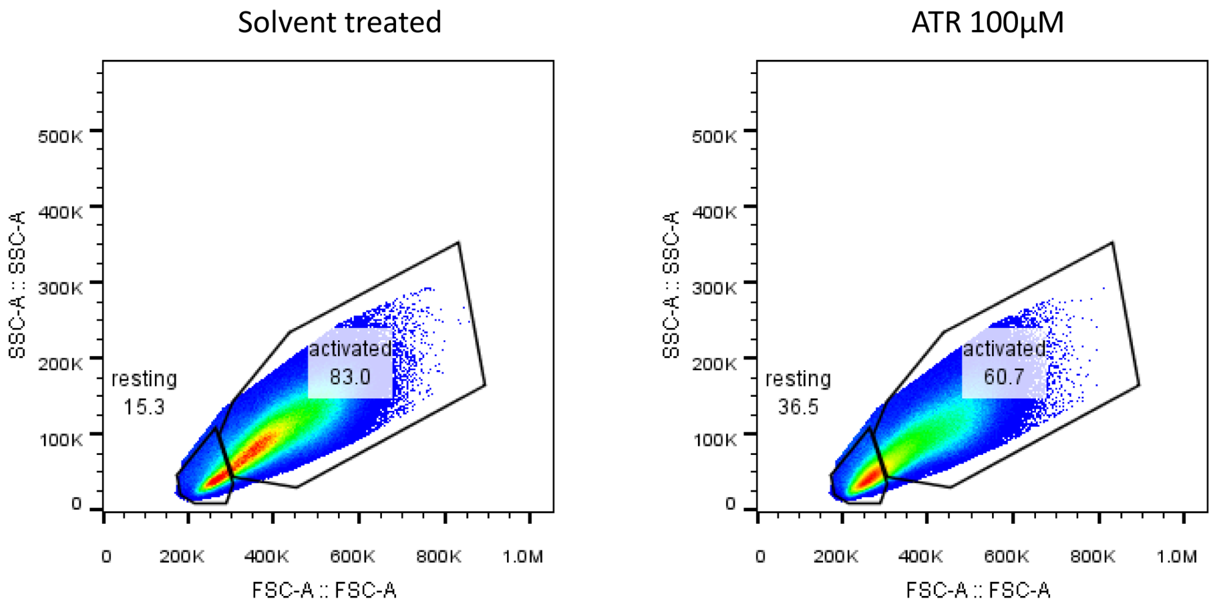

Figure S1. T cell population gated for activated and resting cells.

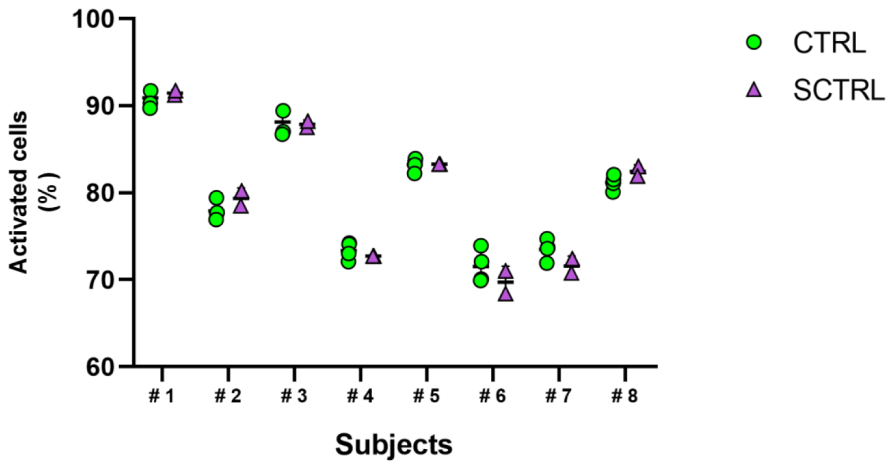

Figure S2. The percentage of activated cells in controls (CTRL) and solvent controls (SCTRL) of each subject. 2-way ANOVA test was used to assess statistical significance of the mean difference between CTRL and SCTRL in each subject. No differences were observed.

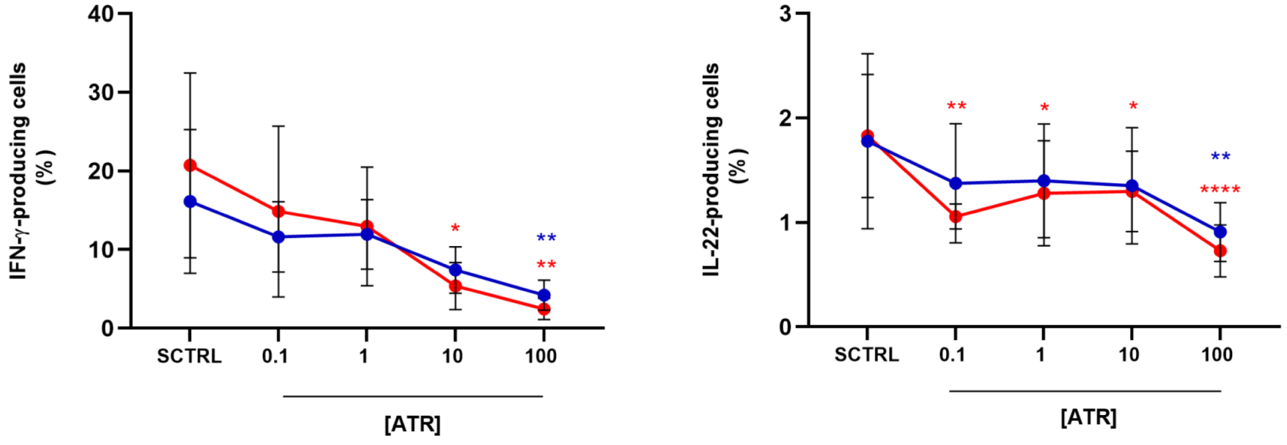

Figure S3. Modulation of IFN-γ- and IL-22-producing cells in male and female subjects. Data from male subjects are shown in blue, and data from female subjects in red. The RM one-way ANOVA test (Friedman) were used to assess statistical significance.

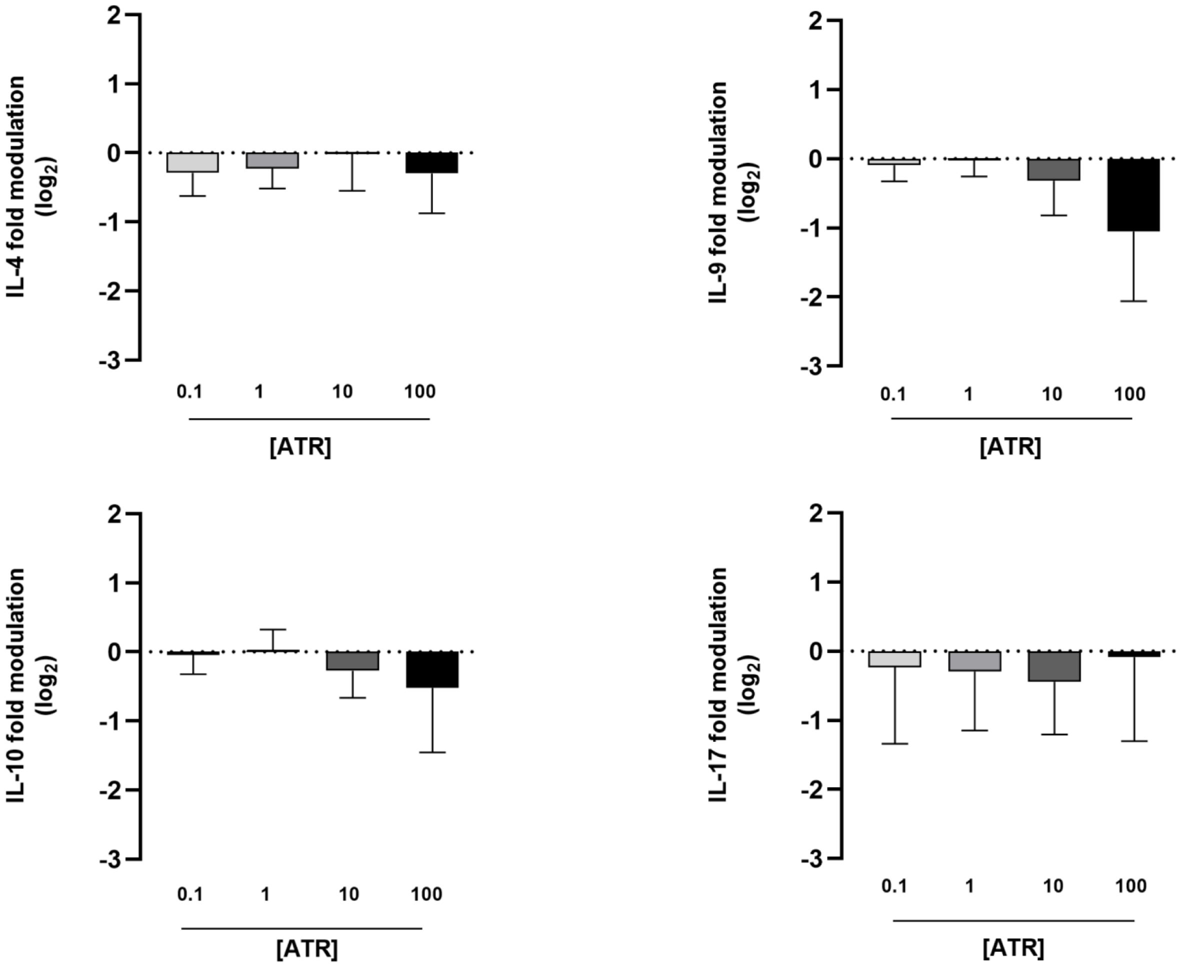

Figure S4. Modulation of cytokine-producing cells (IL-4, IL-9, IL-10, and IL17), following ATR treatment. The mean±SD modulation of the percentage of cytokine producing cells, relative to SCTRL is shown. Kruskal-Wallis test was used to assess statistical significance.

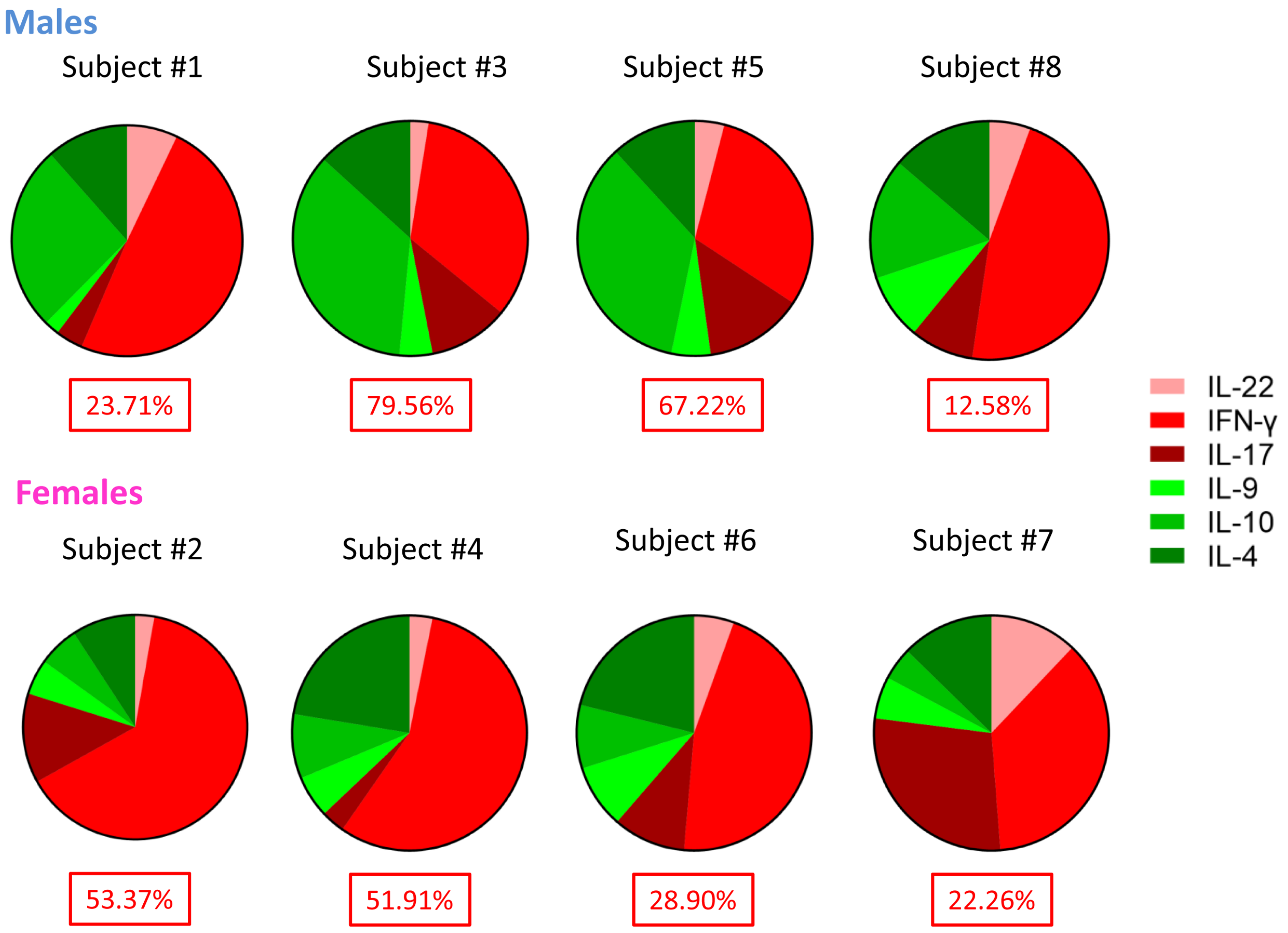

Figure S5. The pie charts show the percentage of cells producing the specified cytokines within the cytokine-producing cells in SCTRL samples from each subject. The percentage of cytokine-producing cells has been reported in the red box.

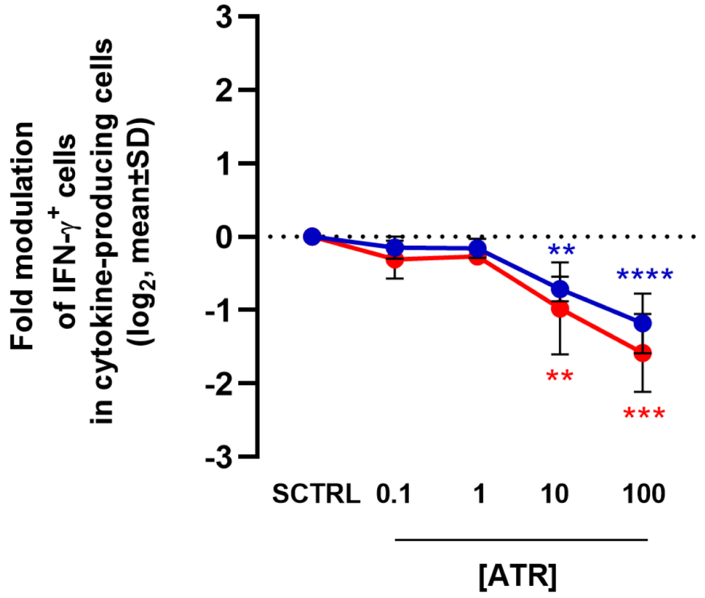

Figure S6. Modulation of IFN-γ-producing cells following ATR treatment in male and female subjects. Data from male subjects are shown in blue, and data from female subjects in red. The RM one-way ANOVA was used to assess statistical significance.

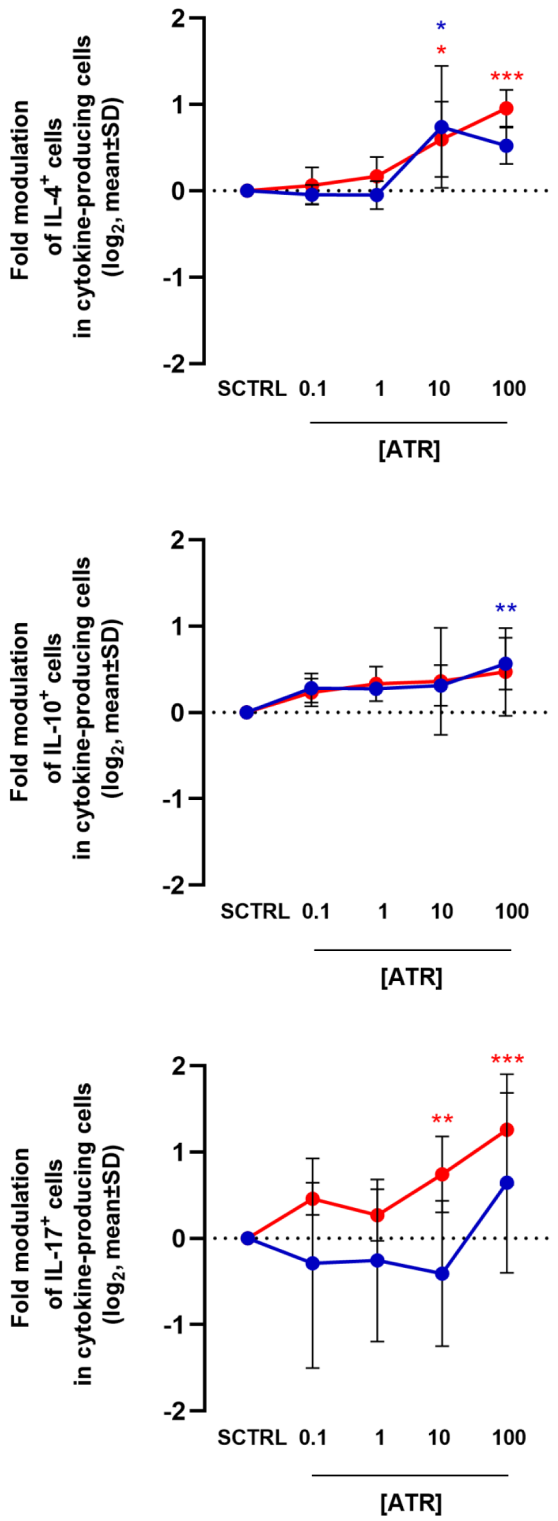

Figure S7. Modulation of IL-4-, IL-10- and IL-17-producing cells in male and female subjects. Data from male subjects are shown in blue, and data from female subjects in red. The RM one-way ANOVA was used to to assess statistical significance.

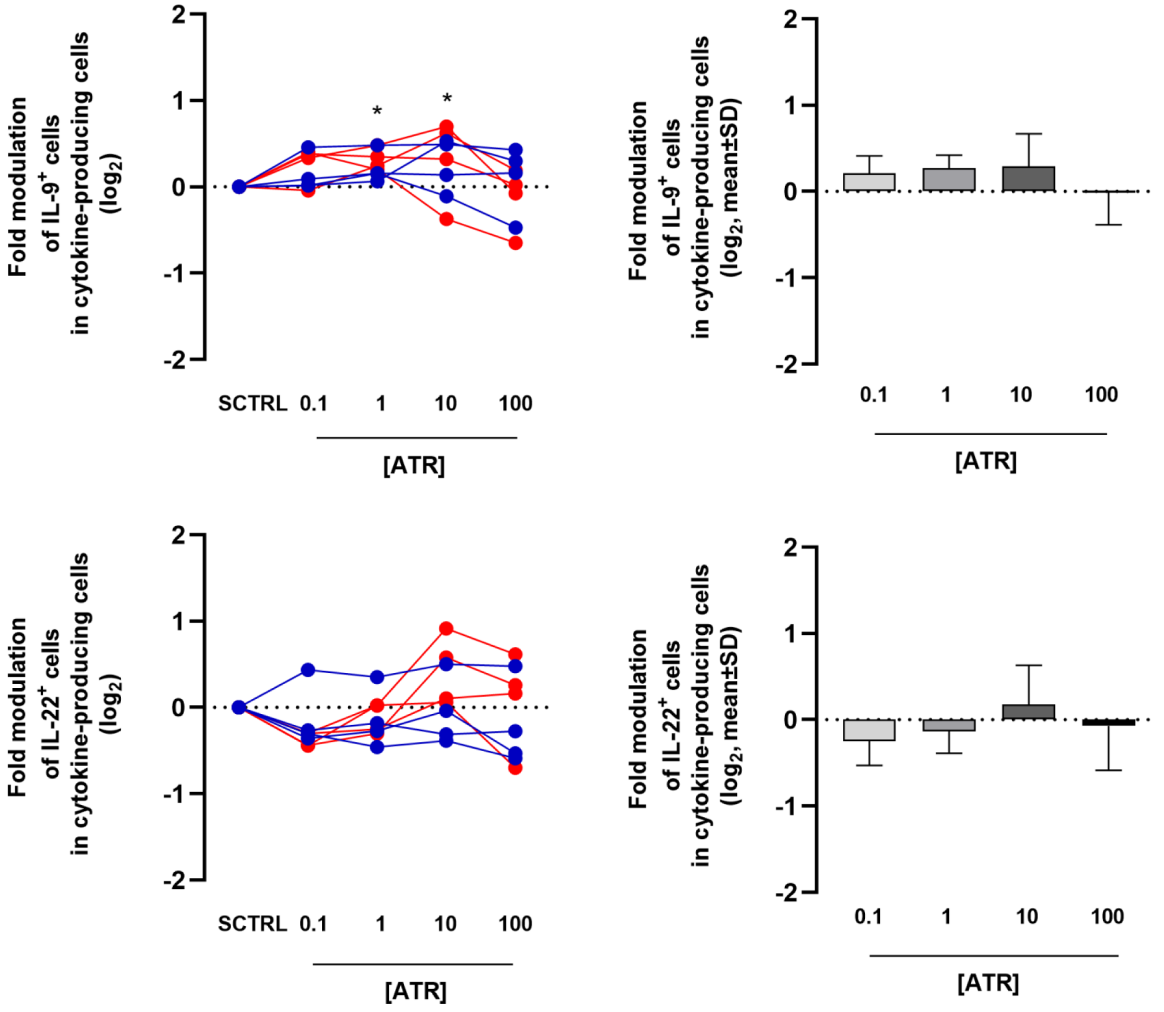

Figure S8. Modulation of IL-9- and IL-22-producing cells in male and female subjects (left panels). Data from male subjects are shown in blue, and data from female subjects in red. The mean±SD modulation of the percentage of cytokine producing cells, relative to SCTRL is also shown (right panels). To assess statistical significance, the Friedman test was used for the dot graphs and the Kruskal-Wallis test was used for the column graphs.

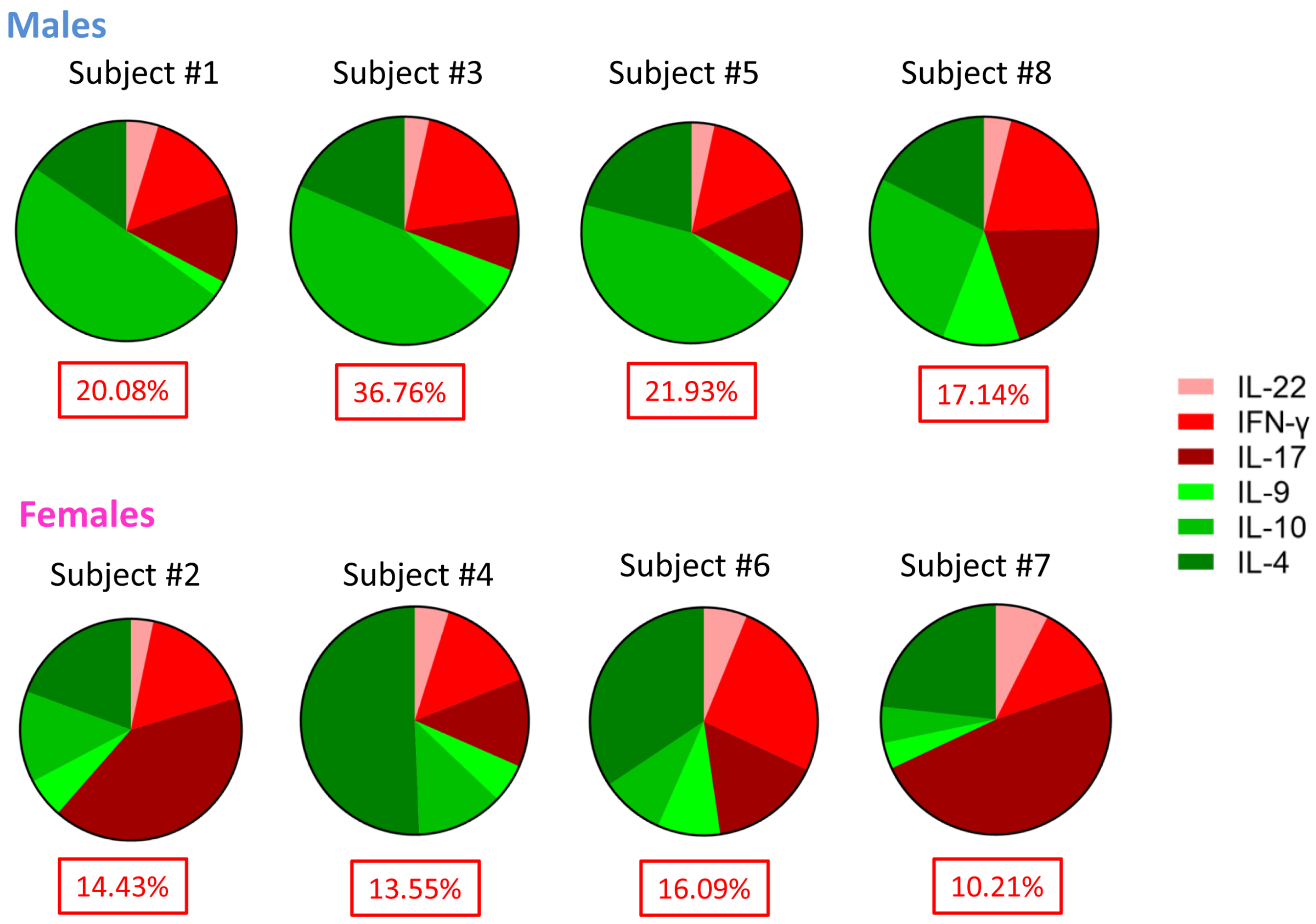

Figure S9. The pie charts show the percentage of cells producing the specified cytokines within the cytokine-producing cells in 100 microM ATR-treated samples from each subject. The percentage of cytokine-producing cells has been reported in the red box.

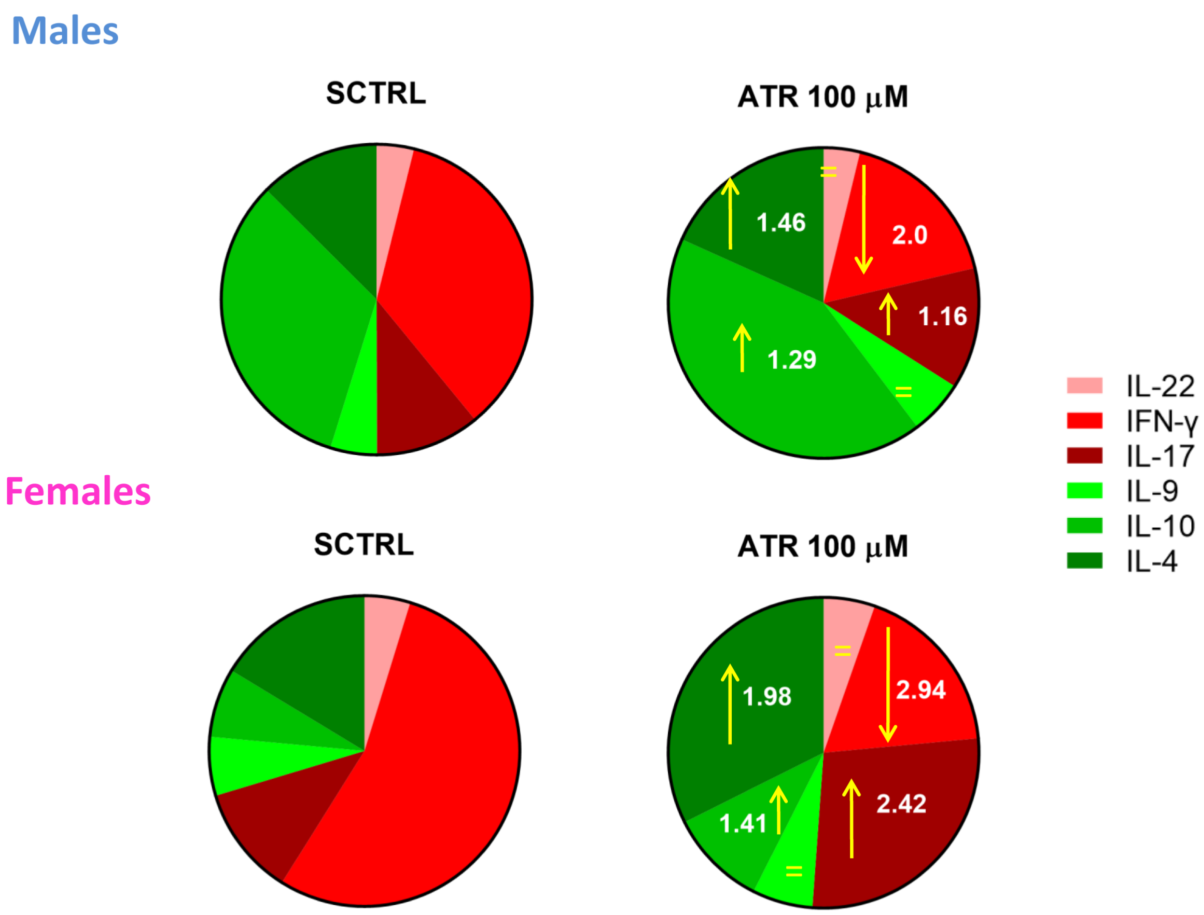

Figure S10. The pie charts illustrate the mean modulation of cytokines within the whole cytokine-producing cells in SCTRL and samples treated with 100 µM ATR in males and females. The arrows pointing upward represents the increase and the ones downwards represents the decrease in modulation. The relative intensity of modulation was shown by shorter or longer size of arrows.

Table S1. Age and sex of donors

Age (years)	Donors	Males/Females
18-24	1	0/1
25-49	6	3/3
50-69	1	1/0

Table S2. Clones and suppliers of antibodies used in the manuscript

Target	Clone	Supplier
CD4	OKT4	Thermo Fisher Scientific
CD8	PRA-TB	Thermo Fisher Scientific
CD25	BC96	Thermo Fisher Scientific
GITR	DT5D3	Miltenyi Biotec
FoxP3	236°/E7	Thermo Fisher Scientific
IFN-γ	4S.B3	Thermo Fisher Scientific
IL-4	BD4-8	Thermo Fisher Scientific
IL-9	MH9D1	Thermo Fisher Scientific
IL-10	JES3-9D7	Thermo Fisher Scientific
IL-17	Ebio64DEC17	Thermo Fisher Scientific
IL-22	IL22JOP	Thermo Fisher Scientific

Table S3. The percentage of cytokine-producing cells in samples from each donor

Donors	sex	SCTRL	0.1 µM	1 µM	10 µM	100 µM
#1	M	23.71	25.84	23.6	22.16	20.08
#3	M	79.56	57.59	61.65	53.57	36.76
#5	M	67.22	43.78	46.87	50.88	21.93
#8	M	12.58	13.48	15.73	11.72	17.14
#2	F	53.37	47.98	40.43	19.37	14.43
#4	F	51.91	40.5	37.81	21.36	13.55
#6	F	28.9	22.35	21.37	22.79	16.09
#7	F	22.26	13.52	16.13	13.41	10.21

Table S4. Modulation of the percentage of cytokine-producing cells in ATR-treated samples as compared to the respective solvent-treated controls, expressed as percentage (positive value = increase, negative value = decrease)

Donors	Sex	0.1 µM	1 µM	10 µM	100 µM
**#1**	M	9%	0%	**-7%**	**-15%**
**#3**	M	**-28%**	**-23%**	**-33%**	**-54%**
**#5**	M	**-35%**	**-30%**	**-24%**	**-67%**
**#8**	M	7%	25%	**-7%**	36%
**Mean***	M	**-12%**	**-7%**	**-18%**	**-25%**
**#2**	F	**-10%**	**-24%**	**-64%**	**-73%**
**#4**	F	**-22%**	**-27%**	**-59%**	**-74%**
**#6**	F	**-23%**	**-26%**	**-21%**	**-44%**
**#7**	F	**-39%**	**-28%**	**-40%**	**-54%**
**Mean****	F	**-24%**	**-26%**	**-46%**	**-61%**
**Mean M/F modulation difference (%)**		12%	19%	28%	36%

* p-value = not significant

**p-value = 0.0091 (0.1 µM), 0.0140 (1 µM), not significant (10 µM), 0.0433 (100 µM)

Table S5. The percentage of CD4$^+$ subpopulation in PBMC of donors after 4-days culture

			atrazine			
**Donor**	**CTRL**	**SCTRL**	**0.1 µM**	**1 µM**	**10 µM**	**100 µM**
**#1**	50.1	50.1	51.0	51.3	51.1	47.6
**#2**	49.5	51.9	50.6	51.1	46.7	44.7
**#3**	54.6	55.3	54.1	53.0	51.8	48.5
**#4**	37.5	37.8	38.6	38.1	38.3	35.4
**#5**	50.1	50.8	51.9	50.7	47.2	45.0
**#6**	59.4	58.4	59.3	58.9	59.4	55.7
**#7**	44.9	44.6	44.3	44.5	43.2	41.3
**#8**	46.3	46.4	45.8	44.8	45.6	43.8

Table S6. Modulation of $CD4^+$ subpopulation percentage vs SCTRL in PBMC of donors, following atrazine treatment after 4-days culture, expressed as percentage (positive value = increase, negative value = decrease)

	atrazine			
**Donor**	**0.1 µM**	**1 µM**	**10 µM**	**100 µM**
**#1**	2%	2%	2%	**-5%**
**#2**	**-3%**	**-2%**	**-10%**	**-14%**
**#3**	**-2%**	**-4%**	**-6%**	**-12%**
**#4**	2%	1%	1%	**-6%**
**#5**	2%	0%	**-7%**	**-11%**
**#6**	1%	1%	2%	**-5%**
**#7**	**-1%**	0%	**-3%**	**-7%**
**#8**	**-1%**	**-3%**	**-2%**	**-6%**
**Mean percentage***	0%	**-1%**	**-3%**	**-8%**

*p-value = not significant (0.1 µM), not significant (1 µM), not significant (10 µM), 0.0007 (100 µM)
